# Importance of Binding Affinity for the Activity of a Metallodendritic Chemical Nuclease

**DOI:** 10.3390/pharmaceutics10040258

**Published:** 2018-12-03

**Authors:** Yi-Hsuan Tang, Sodio C. N. Hsu, Po-Yu Chen, Si-ting Liou, Hui-Ting Chen, Carol Hsin-Yi Wu, Chai-Lin Kao

**Affiliations:** 1 Department of Medicinal and Applied Chemistry, Kaohsiung Medical University, Shih-Chuan 1st Road 100, Kaohsiung 80708, Taiwan; liviatang.tw@gmail.com (Y.-H.T.); sodiohsu@kmu.edu.tw (S.C.N.H.); pyc@kmu.edu.tw (P.-Y.C.); sitingliou@gmail.com (S.-t.L.); 2 Department of Medical Research, Kaohsiung Medical University Hospital, Kaohsiung 80708, Taiwan; 3 Department of Chemistry, National Sun Yat-sen University, 70 Lienhai Rd., Kaohsiung 80424, Taiwan; 4 Department of Fragrance and Cosmetic Science, Kaohsiung Medical University, Shih-Chuan 1st Road 100, Kaohsiung 80708, Taiwan; htchen@kmu.edu.tw; 5 Division of Cellular and Immune Therapy, Department of Medical Research, Kaohsiung Medical University Hospital, Kaohsiung 80708, Taiwan; wuhsinyi2011@gmail.com

**Keywords:** macromolecule nuclease, dendrimers, substrate binding affinity, Cu complexes

## Abstract

A family of *bis*(2-pyridyl)amino-modified poly(amidoamine) dendrimer Cu complexes was prepared, and their chemical nuclease activities and binding affinity (*K_b_*) levels for DNA plasmid were investigated. The *K*_b_ values of the G2 to G6 apodendrimers for DNA plasmid were found to be 7.4, 23, 48, 70, and 280 µM^−1^, respectively, using ethidium bromide (EtBr) displacement experiments. The chemical nuclease activities of the corresponding complexes were determined by gel electrophoresis, and a clear positive dendritic effect was observed. Further analysis indicated a linear correlation between the *K*_b_ values of the G2 to G5 apodendrimers and the nuclease activity of the corresponding complexes. This observation indicated the importance of substrate binding affinity for macromolecular nuclease activity. In addition, an experiment using 3′-(*p*-hydroxyphenyl) fluorescein suggested that hydroxyl radicals formed under the tested conditions. Subsequently performed inhibition studies indicated that the hydroxyl radical was the active species responsible for the plasmid cleavage.

## 1. Introduction

Metallodendrimers have attracted considerable interest in the past few decades because they have the advantages of organic materials while also possessing the properties of metallic ions [1,2,3]. In contrast to small-molecule complexes, metallodendrimers provide unique complexation environments and contain many coordination sites as well as a protective environment for the metallic ions. As a result, metallodendrimers have characteristics not displayed by small-molecule complexes and have been used in various applications [4,5,6,7,8,9].

One representative application of metallodendrimers has been their use as mimics of natural metallomacromolecules, such as metalloenzymes. Metallonucleases are metalloenzymes that contribute to the regulation of the cell cycle by participating in the editing of nucleic acids [10]. In such nucleases, catalytic metallic ions have been shown to function by either of two working mechanisms: hydrolytic or oxidative cleavage. For oxidative nucleases, the coordination environment [11], molecular charge [12], and size of the ligands [13] have been explored, and determined to be vital factors in this catalytic process [14]. Various cofactors, such as H_2_O_2_, are usually necessary to break down nucleic acid chains: these cofactors generate reactive oxygen species during oxidative cleavage by the nuclease [15]. By contrast, hydrolytic nucleases operate using a catalytic mechanism involving assistance by a Lewis acid [16,17]. Although several types of metal ions have been shown to form metallonucleases [18,19], copper ions can act as either Lewis acids or oxidizing agents in nucleases. This unique character makes copper very attractive for the study of both natural and artificial nucleases [20].

In addition to proteins, numerous synthetic molecules, known as chemical nucleases, are capable of excising polynucleic acid backbones, and have been used for biomedical investigations and clinical applications [21,22]. These synthetic analogues of natural metalloenzymes demonstrate programmable nuclease activities and tolerate a wide range of conditions, features necessary for various investigations and applications. Various ligands, therefore, have been developed and characterized [23]. In contrast to aliphatic ligands, aromatic heterocycles can engage in π–π interactions with nucleic acids, and such interactions have been shown to improve the efficiency of the nuclease [24]. Of the various complexes of aromatic heterocycles with metal ions, aminopyridine metal complexes are particularly efficient catalysts due to their structural flexibility [25].

Macromolecular nucleases have been shown to provide advantages over their small-molecule analogues [26]. However, relatively few investigations of macromolecular nucleases have been carried out, due to the tedious nature of preparing monodisperse macromolecules and the difficulty in characterizing their active centers. Both issues have in particular hindered detailed investigations of macromolecular nucleases and their potential biomedical applications. Moreover, while the substrate binding affinities of small-molecule nucleases have been investigated, and reported to be a critical factor for the activity levels of these nucleases [27], the relationship between the activity levels of macromolecular nucleases and their binding affinities for nucleic acids has not been characterized [17] In contrast to other macromolecules, dendrimers are suitable candidates as nucleases because they are monodisperse and well characterized. These characteristic properties endow dendrimers with a well-controlled topology and make them very good models for investigation.

Herein, we report our research results on the use of metallodendrimers as chemical nucleases. G2–G6 poly(amidoamine) (PAMAM) dendrimers with peripheral *bis*(2-pyridyl)amino groups were synthesized and then coordinated with copper (Cu) ions to obtain nuclease complexes. These Cu complexes were tested for their nuclease activities, and the results were analyzed to reveal the relationship between the metallodendrimer nuclease activity and binding affinity to DNA.

## 2. Materials and Methods

### 2.1. DNA Binding Affinity

Ethidium bromide (EtBr) displacement assay studies were used to measure the binding affinities between dendrimer **2a** to **2e** and CT-DNA. The CT-DNA solution (0.46 mM) and ethidium bromide (EtBr, TCI, Tokyo, Japan) (50 µM) were dissolved in buffer (tris buffer 24 mM, pH = 7.24) and mixed in a florescence cuvette. A series of experiments were conducted by adding 0–14 µM of the compounds **2a**–**2e** individually to the EtBr-bound CT-DNA in the florescence cuvette, and the florescence intensities were measured over time. The normalized florescence intensity was then plotted against the dendrimer concentration.

### 2.2. Nuclease Activity

The cleavage activity was investigated by agarose gel electrophoresis. Complexes **3a** (1.67 µM, 3.33 µM, 6.67 µM, 8.33 µM, 10.00 µM, 16.67 µM, 20.00 µM) were mixed with plasmid DNA in the presence of 1,4-dithiothreitol (DTT) (0.66 mM) in Tris buffer (pH = 7.24, 24 mM), and the fluorescence was monitored over 45 min. Thereafter, the plasmid DNA was stained with EtBr and separated on a 0.8% agarose gel in standard Tris/borate /EDTA (TBE) buffer at 110 V over 35 min. The fluorescence intensity of form II under various conditions was obtained by using the ImageJ software.

### 2.3. Reactive Oxygen Species (ROS) Responsible for DNA Cleavage

Radical scavengers or metal chelators, such as sodium formate (53 mM), neocuproine (0.53 mM), EDTA (13 mM), and sodium azide (53 mM), were treated with **5e** (33 nM) and DTT (0.66 mM). Complex **5e** was mixed with DTT and various radical scavengers and incubated for 2 h. The solution was then separated on a 0.8% agarose gel in the standard Tris/borate/EDTA (TBE) buffer at 110 V over 35 min.

### 2.4. Determination of Hydroxyl Radicals

Tris buffer (24 mM, pH = 7.24), was degassed by bubbling nitrogen through the solution over two h to form an anaerobic environment. Compound **5e** chelated 57 Equations copper ions to form a copper complex. The copper complex (2.60 µM) and DTT (161.00 µM) were mixed in the degassed Tris buffer and transferred to a fluorescence cuvette. H_2_O_2_ (0.27 mM, TCI) and 3′-(*p*-hydroxyphenyl) fluorescein (HPF) dye (Thermo Fisher, Waltham, MA, USA) were added to the fluorescence cuvette to obtain the reduced copper complex. The fluorescence intensity was used to determine the presence of hydrolytic radicals.

## 3. Results and Discussion

**Scheme 1:**
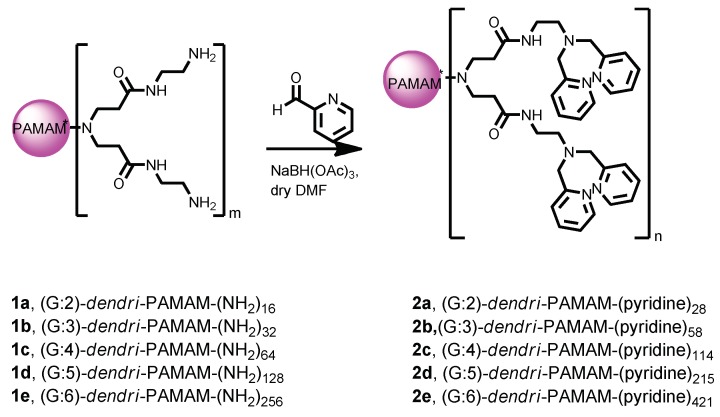
Synthesis of compounds **2**.

**Figure 1:**
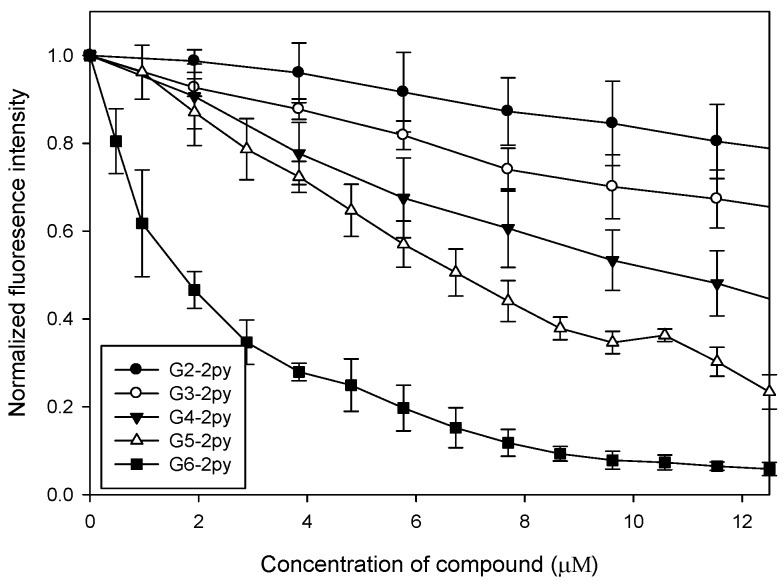
Ethidium bromide (EtBr) displacement assay.

The *bis*(2-pyridyl)amino-modified (PAMAM) dendrimers (**2**) were prepared according to the literature [28] (Scheme 1). To study their binding affinity, an ethidium bromide (EtBr) displacement assay was used and the results indicated a clear negative correlation between the fluorescence intensity of EtBr and the concentration of each of the synthetic dendrimers **2**. For any given quantity of the dendrimer, the high-generation dendrimers displayed lower fluorescence intensity levels than the low-generation ones. This observation suggested that the high-generation dendrimers bound more strongly to the nucleic acids (Figure 1). From these data we computed binding constants of 7.4, 23, 48, 70, and 280 µM^−1^ for **2a**–**2e**, respectively [19], i.e., the larger the dendrimer, the stronger its binding of the nucleic acid.

**Figure 2:**
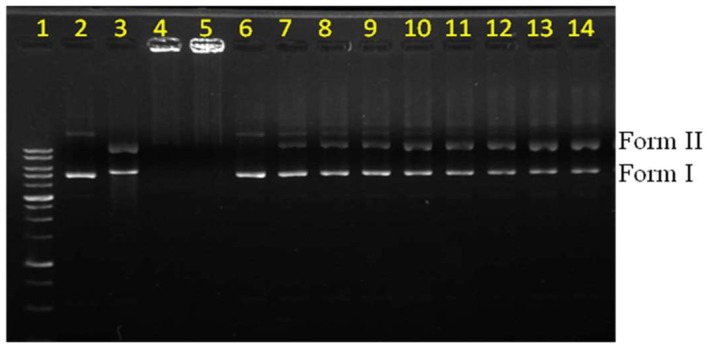
Electrophoresis experiments were performed to characterize the conditions under which reactive oxygen species (ROS) radicals were generated and nuclease activity was observed. Lane 1: marker; Lane 2: DNA; Lane 3: bleomycin (10.00 µM); Lane 4: CuSO_4_ (1.6 mM) + 4 (16.7 µM) + DTT (0.66 mM); Lane 5: CuSO_4_ (1.6 mM) + DTT (0.66 mM); Lane 6: **3a** (1.67 µM); Lane 7: DTT (0.66 mM); Lane 8: **3a** (1.67 µM) + DTT (0.66 mM); Lane 9: **3a** (3.33 µM) + DTT (0.66 mM); Lane 10: **3a** (6.67 µM) + DTT (0.66 mM); Lane 11: **3a** (8.33 µM) + DTT (0.66 mM); Lane 12: **3a** (10.00 µM) + DTT (0.66 mM); Lane 13: **3a** (16.67 µM) + DTT (0.66 mM); Lane 14: **3a** (20.00 µM) + DTT (0.66 mM). DTT = 1,4-dithiothreitol.

To investigate the nuclease activity, we first saturated dendrimer **2a** with Cu and then subjected the resulting complex **3a** to a DNA cleavage experiment. **3a** displayed nuclease activity toward the supercoiled form (form I) of the plasmid to produce the circular form (form II) in the presence of 1,4-dithiothreitol (DTT) as a reducing agent (Figure 2). In this investigation, bleomycin was used as a reference compound (lane 3). Different amounts of the complex (**3a**) were used to study its nuclease activity, and the results are indicated in lanes 7–14. The quantity of the circular form (form II) of the DNA plasmid increased in the presence of larger quantities of the complex. The concentration-dependent nuclease activity was used to characterize the catalytic efficiency of this complex. Complex **3a** alone showed no nuclease effect (lane 6). This observation implied that no role was played by the hydrolytic mechanism in this reaction. To understand the possible effect of ligand and copper alone, a comparable amount of bipyridine analogue (**4**, Figure S1) and CuSO_4_ were subjected to similar experiments and the results are shown in lanes 4 and 5; here the plasmid remained in the loading well. Presumably, the negative charges of the plasmid were neutralized by the excess quantity of copper ions, making the complex stationary on the gel. The monomeric dendrimer and the free copper ions have been reported to show no ROS generation activity under the given conditions [28]. Therefore, the monomer was assumed to have no nuclease activity in the present studies. The observed activity was therefore concluded to have arisen from the macromolecular Cu complex rather than from the free Cu ions in the medium.

**Figure 3:**
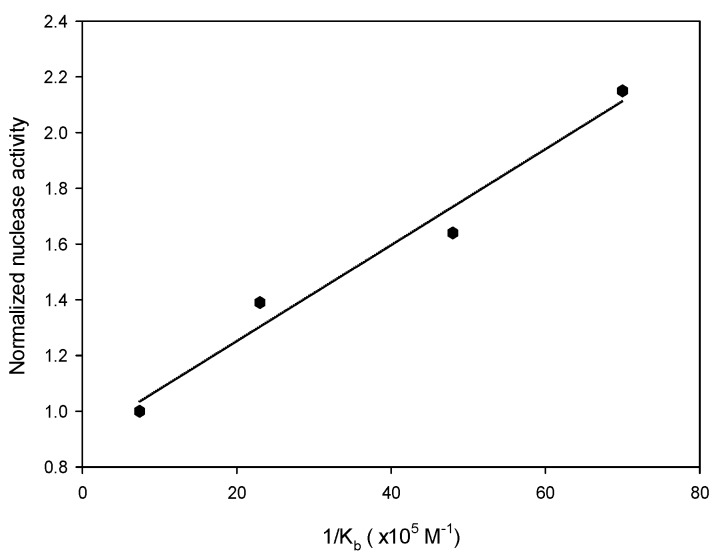
Plot of the normalized nuclease activities of hexaCu complexes **5a**–**5d** as a function of 1/*K_b_* for apodendrimers **2a**–**2d** toward the plasmid. The fluorescence intensity of form II in the presence of each compound **5** was obtained by using ImageJ software.

To explore the relationship between dendrimer binding affinity and nuclease activity, the number of Cu ions in each dendrimer must be the same to prevent any possible bias associated with a different number of Cu ions in each dendrimer. In previous work [29], each G2-dendrimer (**2a**) molecule was found to bind to no more than six copper ions. Accordingly, six equivalents of copper ions were coordinated to each dendrimer generation (**2a**–**2e**) to form hexaCu complexes **5a**–**5e**, respectively, and these complexes were subjected to nuclease activity assays. The fluorescence intensities obtained from form II indicated that the nuclease activity was positively correlated with the dendrimer size (Figure S2). High-generation dendrimers exhibited stronger plasmid DNA cutting activities than did the low-generation analogues. Dendrimers **5d** and **5e** exhibited, respectively, 2.15-fold and 2.02-fold higher nuclease activities than did **5a**. Meanwhile, **5b** and **5c** exhibited, respectively, only 1.39-fold and 1.64-fold higher nuclease activities than did **5a**. Surprisingly, the normalized nuclease activities of metallodendrimers G2 through G5 were found to be highly positively correlated (*R*^2^ = 0.97) with the *K_b_* values of the corresponding apodendrimers for the DNA plasmid (Figure 3). These results indicated that the binding affinity of the dendrimer for the DNA plasmid directly contributed to its nuclease activity. While **5e** was expected, based on the trend of the results for **5a**–**5d**, to exhibit higher activity than **5d,** it was obser ved to display only 94% of the activity towards form II of **5d**. (Table S1) This observation may have been due to the greater degradation of form II in the presence of complex **5e**.

Identifying the active species responsible for the nuclease activity is also an important issue for developing biomedical applications of the nuclease. To determine the major active species of the synthesized dendrimers responsible for their cleavage of plasmid, we only considered oxygen-derived species, since hydrolytic cleavage is not possible here [26]. Of the various possible reactive oxygen species (ROS), the hydroxyl radical has been found to be responsible for the activities of various nucleases [27]. Therefore, the hydroxyl radical was hypothesized to be involved in the activity of the dendrimer nuclease. This hypothesis was tested by determining the presence of hydroxyl radicals using 3′-(*p*-hydroxyphenyl) fluorescein (HPF), which reacts predominantly with hydroxyl radicals or superoxide anion radicals via dearylation to form a fluorescent product. Superoxide, however, can induce re-oxidation reactions that disturb the identification of the hydroxyl radical. Tris buffer was, therefore, degassed to avoid the formation of metallodendrimer-generated superoxide anion radicals in the aerobic environment used. Instead, H_2_O_2_ was used as the oxygen source and mixed with **5e** in an anoxic environment, and the formation of hydroxyl radicals was monitored using HPF. The fluorescence intensity was much higher in the presence of the metallodendrimer than in the absence of the metallodendrimer (Figure S3). This observation using complex **5e** indicated that this dendrimer, and hence likely **5a**–**5d** as well, can catalyze the formation of the hydroxyl radical.

**Figure 4:**
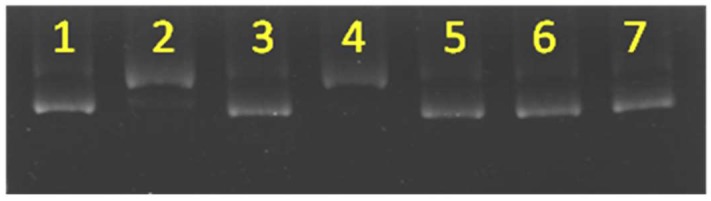
Identification of the active species. Lane1, plasmid alone; Lane 2, plasmid with **5e** (33 nM) + DTT (0.66 mM); Lane 3, plasmid with **5e** (33 nM) + DTT (0.66 mM) + sodium formate (53 mM); Lane 4, plasmid with **5e** (33 nM) + DTT (0.66 mM) + neocuproine (0.53 mM); Lane 5, plasmid with **5e** (33 nM) + DTT (0.66 mM) + EDTA (13 mM); Lane 6, plasmid with **5e** (33 nM) + DTT (0.66 mM) + sodium azide (53 mM); Lane 7, plasmid with **5e** (33 nM).

To further confirm the presence of the hydroxyl radical under the given conditions, we carried out inhibition studies using various ROS scavengers or metal chelators, including EDTA, sodium azide, neocuproine, and sodium formate, which served as a copper chelator, singlet oxygen inhibitor/copper chelator, specific copper (I) chelator, and hydroxyl radical scavenger, respectively. The inhibition activities of these compounds were examined by introducing each inhibitor into the DNA cleavage experiments and measuring the resulting nuclease activities. As shown in Figure 4, in lanes 3, 5, and 6, the plasmid remained in form I when sodium formate, EDTA, and sodium azide, respectively, were included, clearly indicating that each of them inhibited DNA cutting (Figure 4). EDTA removed metal ions from the dendrimers. The nuclease activity results indicated that the copper ions were crucial to the nuclease activity. Also note that azide has been shown to chelate copper ions and abrogate copper re-oxidation in catecholases and tyrosinases [30]. Moreover, sodium azide could also act as a singlet oxygen scavenger. However, the presence of singlet oxygen was highly unlikely in this reaction, as it did not involve photo-irradiation. The presence of reducing agents was necessary for the nuclease activity, and sodium azide appeared to act as a copper chelator but not a singlet oxygen scavenger in this reaction. Nuclease inhibition by sodium formate implied that the hydroxyl radical was the ROS involved in DNA cleavage. Nevertheless, the failure of neocuproine to remove the copper(I) ions and hence stop the reaction may have been due to the strong binding of Cu(I) ions to the *bis*(2-pyridyl)amino moieties, indicating that neocuproine could not remove the copper(I) ions or stop the reaction.

**Figure 5:**
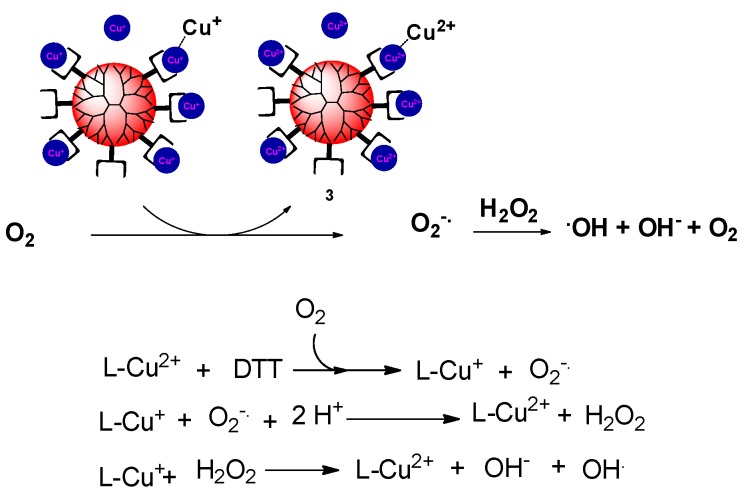
Hypothetical mechanism for the dendrimer-induced production of hydroxyl radicals.

In view of previous studies [31], the Cu dendrimer complexes apparently generated superoxide anion radicals in the presence of reducing agents. The superoxide anion radicals could be then further reduced and protonated to form hydrogen peroxide. The hydroxyl radical could have been obtained from either one of two routes. With excess reducing agent, Cu^+^ ion conjugated to hydrogen peroxide may have produced a highly reactive hydroxyl radical through the copper Fenton reaction. Otherwise, the Haber–Weiss reaction might have been involved while reductant was being consumed. Under this circumstance, Cu^2+^ was reduced by superoxide anion radicals to give the Cu^+^ ion, which could lead to the formation of the hydroxyl radical (Figure 5).

## 4. Conclusions

The most significant discovery of this investigation was the establishment of a correlation between the binding affinity of copper dendrimer-based nucleases and their nuclease activity. For the first time, a linear correlation between *K_b_* and chemical nuclease activity was found among the complexes tested. The higher-generation dendrimers displayed higher binding affinities for the DNA plasmid, and the nuclease activity of the dendrimer complex was found to depend on the size of the dendrimer. These results revealed the importance of binding affinity for macromolecular nuclease activity and could be used for the development of new macromolecular nucleases. We also identified the formation of hydroxyl radicals to be the major working mechanism for this cleavage reaction. Our observations here have provided a fundamental understanding of how macromolecules can serve as nucleases and pave the way for the design of efficient chemical nucleases.

## Supplementary Materials

The following are available online at http://www.mdpi.com/1999-4923/10/4/258/s1, Procedures used to synthesize compounds **2** and **4,** complexes **3a** and **5**; H-NMR spectra of compounds **2**. Figure S1. Structure of compound **4**; Figure S2. Nuclease activity of hexaCu complexes **5**; Figure S3. Fluorescence spectra of HPF in the presence and absence of **5e**; Table S1. Relative fluorescence intensity of II.
