# Supplementary material for: Importance of Binding Affinity for the Activity of a Metallodendritic Chemical Nuclease
Source: Pharmaceutics. 2018 Dec 3;10(4):258. doi: 10.3390/pharmaceutics10040258 (PMC6320806; doi:10.3390/pharmaceutics10040258)
Supplement: Supplementary file 1 [file pharmaceutics-10-00258-s001.pdf]

pharmaceutics

# Supplementary Materials: Importance of Binding Affinity for the Activity of a Metallodendritic Chemical Nuclease

**Yi-Hsuan Tang, Sodio C. N. Hsu, Po-Yu Chen, Si-ting Liou, Hui-Ting Chen, Carol Hsin-Yi Wu and Chai-Lin Kao**

## General Information

Chemicals were purchased from Sigma-Aldrich, Acros, and Merck. Co. PAMAM dendrimers were purchased from Dendritech Co. Flash chromatography was performed on a 230–400 mesh silica （Silica Gel 60） from Merck. Co. NMR spectra were obtained on a Varian 400 MHz spectrometer. MALDI-Mass spectra were recorded using an Autoflex III MALDI-TOF system (Bruker Daltonics). UV-Vis spectra were collected using a JASCO V500 spectrophotometer. Florescence spectra were recorded using a Cary Eclipse fluorescence spectrophotometer. Gel electrophoresis experiments were conducted using a Major Science MP-500P programmable electrophoresis power supply and a UVP Biospectrum.

## Synthesis of compounds 2 and 4; complexes 3a and 5:

(G:2)- *dendri*-PAMAM- $Py_{28}$ (**2a**).

According to previous literature[i], product **2a-2e** were prepared.$^1$H-NMR (400 MHz, $D_2O$) δ: 8.64 (d, *J* = 5.2 Hz, 28 H), 8.43 (t, *J* = 16 Hz, 28 H), 7.98 (d, *J* = 8.0 Hz, 28 H), 7.88 (t, *J* = 13.2 Hz, 28 H), 4.21(s, 49 H), 3.52~3.29 (m, 143 H), 2.79~2.70(m, 81 H). Mass (MALDI, m/z) cacld.: M: 5804; $(M+2H^+)/2$: 2903 Found: 5806; 2903

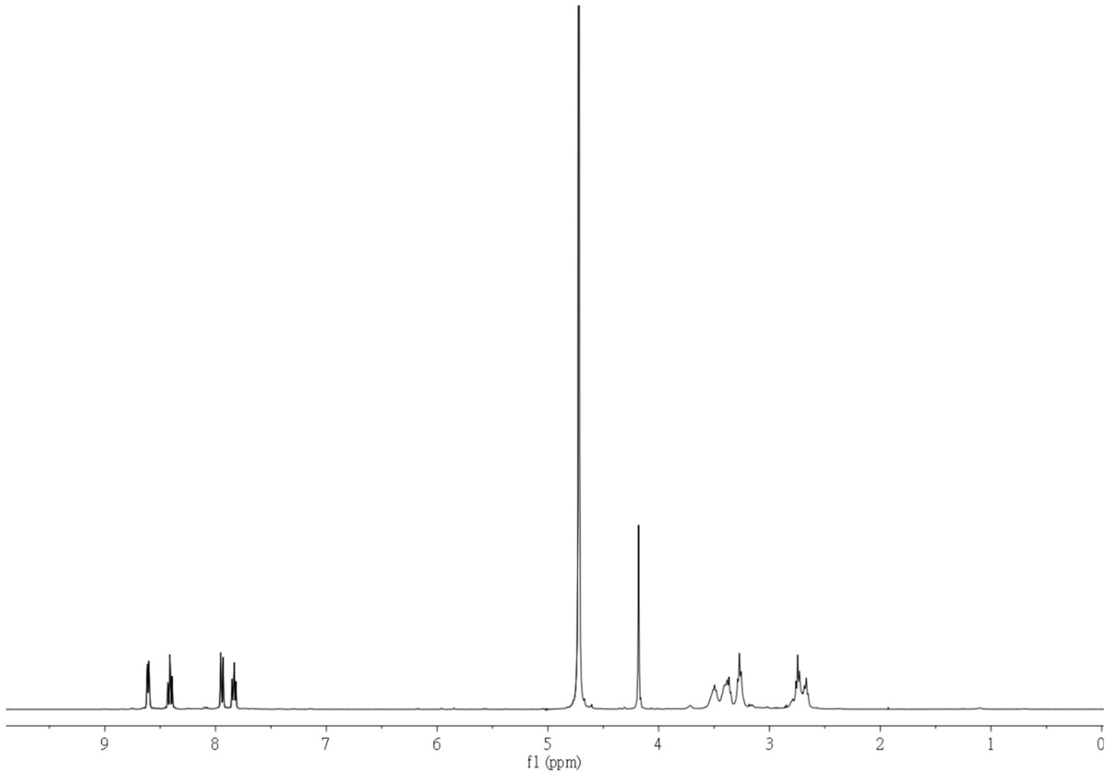

(G:3)-PAMAM-*dendri*-Py$_{57}$ (**2b**).

$^{1}H$-NMR (400 MHz, $D_2O$) δ: 8.59 (d, *J* = 6 Hz, 57 H), 8.40 (t, *J* = 15.6 Hz, 58 H), 7.92 (d, *J* = 8.0 Hz, 57 H), 7.80 (t, *J* = 13.6 Hz, 58 H), 4.15 (s, 108 H), 3.48~3.23 (m, 309 H), 2.73~2.64 (m, 170 H). Mass (MALDI, m/z) cacld.: M: 12187; (M+ $3Na^+$+$3K^+$)/6: 2061; (M+$3H^+$)/3: 4063. Found: 2061; 4063

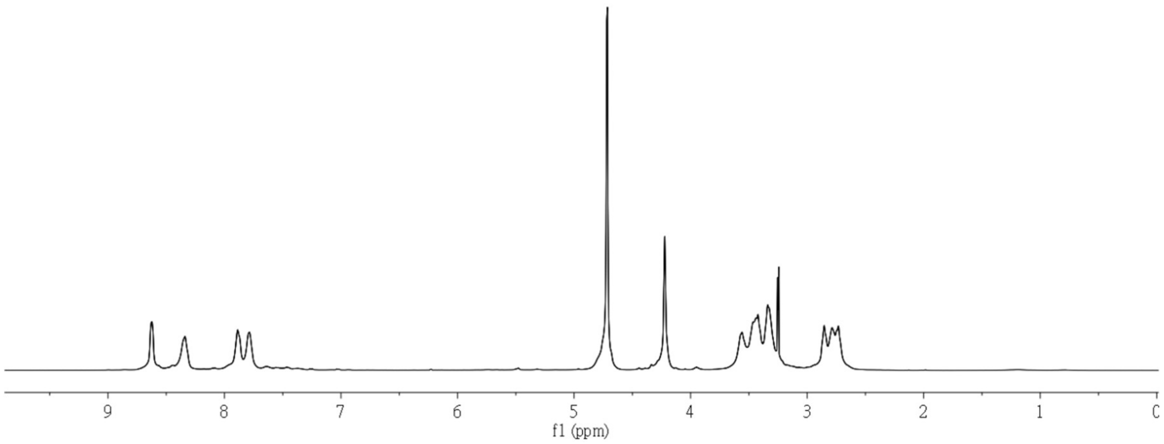


(G:4)-PAMAM-*dendri*-Py$_{114}$ (**2c**).

$^{1}H$-NMR (400 MHz, $D_2O$) δ: 8.68 (d, *J* = 6 Hz, 89 H), 8.46 (b, 100 H), 8.00 (d, *J* = 8 Hz, 102 H), 7.88 (b, 102 H), 4.26 (s, 173 H), 3.59~3.27 (m, 663.0 H), 2.84~2.75 (m, 335 H). Mass (MALDI, m/z) cacld.: M: 24225 ; (M+ $Na^+$ + 12 $H^+$)/13: 1866. Found: 1866

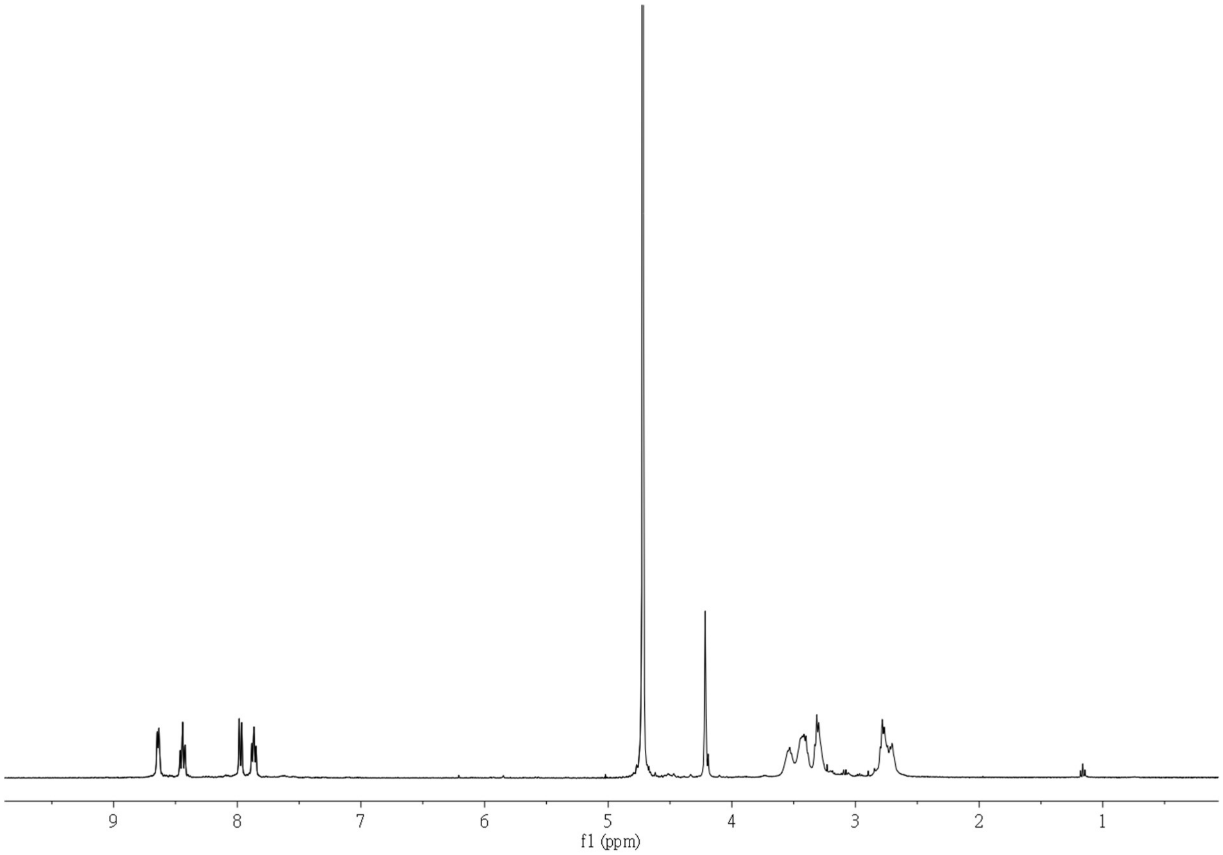

(G:5)-PAMAM-*dendri*-$Py_{215}$ (**2d**).

$^1$H-NMR (400 MHz, $D_2O$) δ: 8.69 (d, *J* = 5.6 Hz, 187 H), 8.46 (b, 202 H), 8.00 (d, *J* = 8.0 Hz, 204 H), 7.89 (b, 202 H), 4.26 (s, 335 H), 3.57~3.27 (m, 1325 H), 2.84~2.75 (m, 700 H). Mass (MALDI, m/z) cacld.: M: 48391; (M+ 4 $Na^+$ + $27H^+$)/31: 1565; (M+ 28 $H^+$)/28: 1729; (M+ 23 $H^+$)/23: 2105. Found: 1565; 1729; 2105

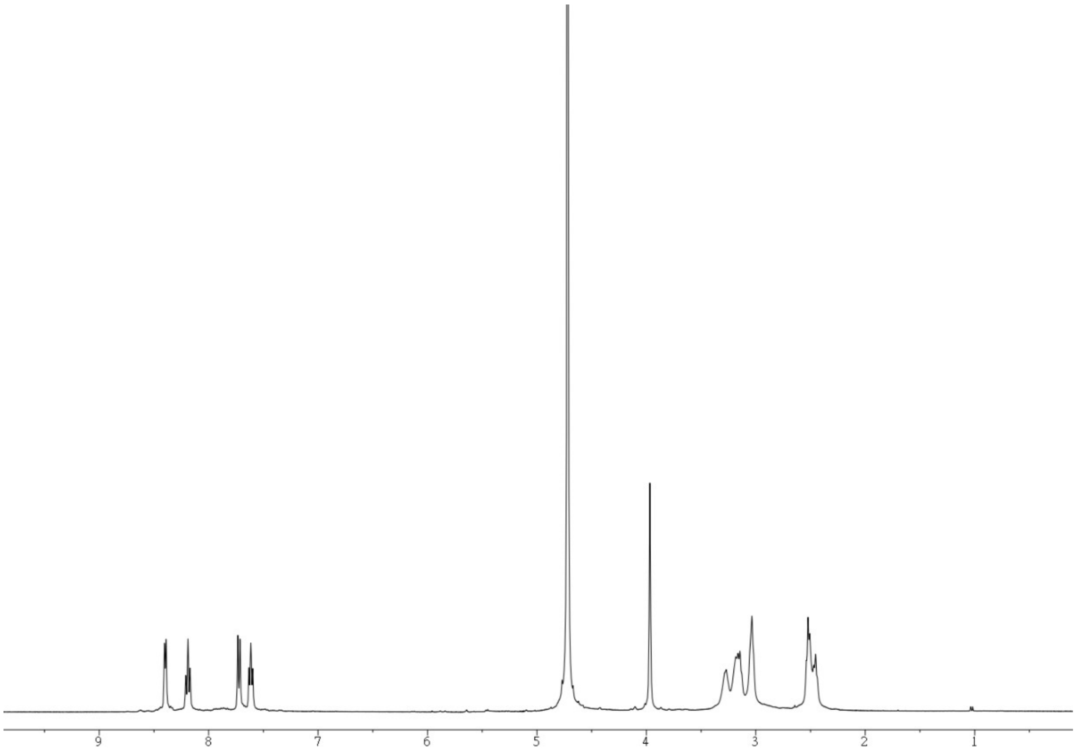


(G:6)-PAMAM-*dendri*-$Py_{421}$ (**2e**).

$^1$H-NMR (400 MHz, $D_2O$) δ: 8.60 (d, *J* = 5.6 Hz, 421 H), 8.41 (t, *J* = 8 Hz, 421 H), 7.93 (d, *J* = 8 Hz, 421H), 7.83 (t, *J* = 6.4 Hz, 421H), 4.18 (s, 842 H), 3.48~3.25 (m, 2648 H), 2.75~2.66 (m, 1420 H). Mass (MALDI, m/z) cacld.: M: 1439.5 (M + $Na^+$ + $66H^+$ )/67, 3027.2 (M + $14Na^+$ + $5K^+$ + $13H^+$ )/32, 1347.4 (M + $12Na^+$ + $9K^+$ + $51H^+$ )/72; Found: 1439.5; 3027.2; 1347.5.

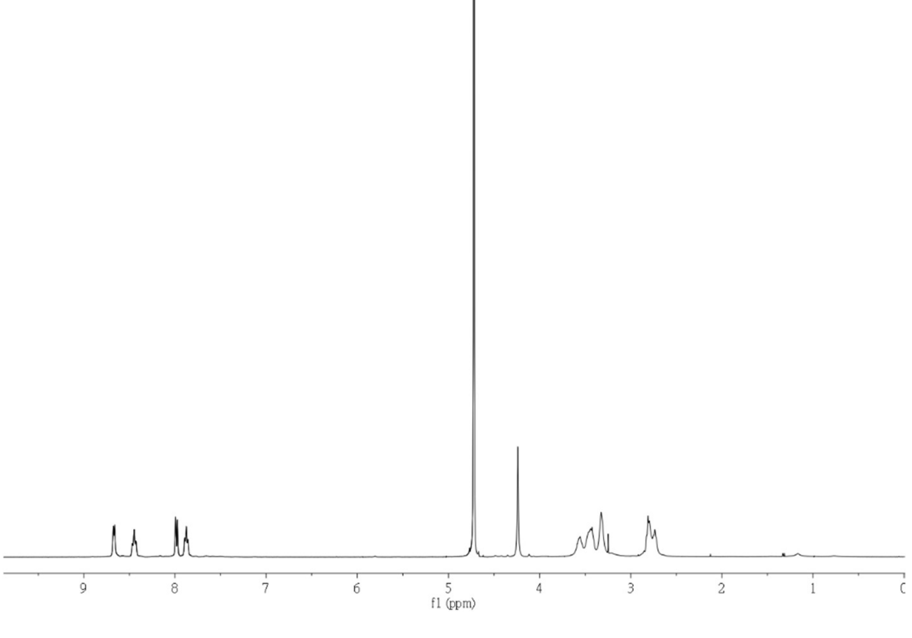

**Preparation of Complexes 3a:**

To the solution of compound **2a** in methanol was added the solution of $CuSO_4 \cdot 5H_2O$ in methanol dropwise to give precipitate. After centrifuge, the solvent was removed to give desired product as blue green solid (**3a**).

**Synthesis of *N,N*-bis(2-pyridylmethyl)-3-aminopropanol (4):**

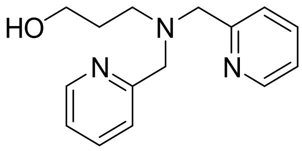

**4**

**Figure S1.** Structure of compound **4**.

To a mixture of 3-amino-1-propanol (0.5 g, 6.7 mmol) and pyridine- 2-carboxaldehyde (1.5 g, 14 mmol) in dry dichloromethane (20 mL) was added sodium triacetoxyborohydride (2.96 g, 14 mmol) under $N_2$. After being stirred for 48 h under a $N_2$, dichloromethane was removed. The resulting mixture was dissolved in chloroform and washed with aqueous solution. The product was purified by chromatography over silica gel (eluting with a 20/1 mixture of chloroform-methanol) to give a pale yellow oil (0.94g, 3.7 mmol, yield 55%, Rf= 0.3 )$^1$H NMR (400 MHz, $CDCl_3$) δ: 8.56–8.54 (m, 2H), 7.66–7.61 (m, 2H), 7.42–7.41 (d, *J* = 4.0 Hz, 2H), 7.18–7.14 (m, 2H), 3.84 (s, 4H), 3.73 (t, *J* = 10.4 Hz, 2H), 2.78 (t, *J* = 11.6 Hz, 2H), 1.83-1.78 (m, 2H). Mass (EI-MS m/z) cacld: M: 257.1528. Found: 257.1530

**Preparation of hexaCu complexes 5a~e:**

Copper sulfate solution (1 mM; 6 µl, tris buffer) was mixed with compound (**5a** to **5e**) (1 mM; 1 µl, tris buffer) solution to form complexes **5a** to **5e.**

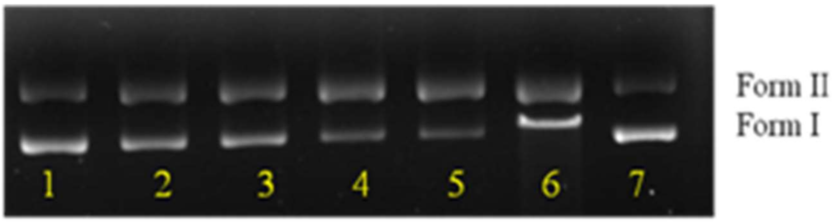


**Figure S2.** Nuclease activity of HexaCu complexes **5.** DNA cleavage activity of **5**. Lane 1, compound **5a** (33 nM) + DTT (0.66 mM); lane 2, compound **5b** (33 nM) + DTT (0.66 m**M**); lane 3, compound **5c** (33 nM) + DTT (0.66 mM); lane 4, compound **5d** (33 nM) + DTT (0.66 mM); lane 5, compound **5e** (33 nM) + DTT (0.66 mM); lane 6, bleomycin; lane 7, Tris buffer (24 mM).

**Table S1.** Relative fluorescence intensity of from II .

| compounds | 3a | 3b | 3c | 3d | 3e |
|---|---|---|---|---|---|
| relative activity | 1.00 | 1.39 | 1.64 | 2.15 | 2.02 |

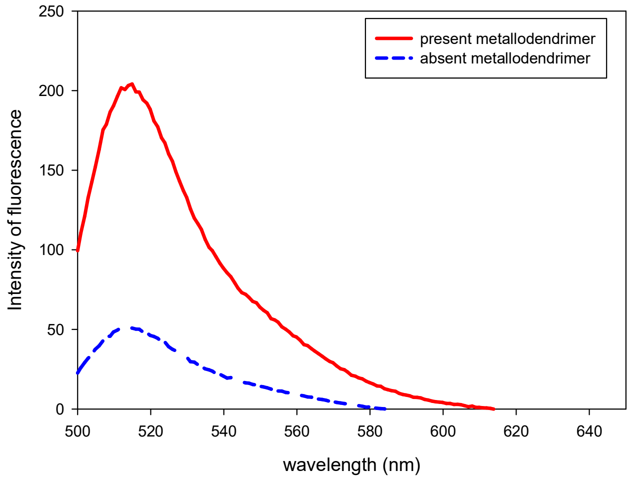


**Figure S3.** Fluorescence spectra of HPF in the presence or absence of **5e**.

---

[i] Kao, C.-L.; Tang,Y-h; Lin, Y.C ; Chiu, L.-T.; Chen, H.-T.; Hsu, S.C.N.; Hsieh,K.-C.; Lu, C.-Y.; Chen, Y.-L. *Nanomedicine, NBM.* **2011**, *7*, 273–276.
